# Asymptomatic and Symptomatic COVID-19 Infections Among Health Care Personnel Before and After Vaccination

**DOI:** 10.1001/jamanetworkopen.2021.15980

**Published:** 2021-07-08

**Authors:** Shruti K. Gohil, Keith Olenslager, Kathleen A. Quan, Cyrus K. Dastur, Nasim Afsar, Wayne Chang, Susan S. Huang

**Affiliations:** 1 Epidemiology & Infection Prevention Program, University of California, Irvine Health (UCI Health), Irvine; 2 Division of Infectious Diseases, University of California, Irvine School of Medicine, Irvine; 3 Division of Neurocritical Care, Department of Neurology, University of California, Irvine School of Medicine, Orange; 4 UCI Health, Los Angeles, California; 5 Division of Occupational and Environmental Medicine, University of California, Irvine School of Medicine, Irvine

## Abstract

This cohort study investigates asymptomatic and symptomatic COVID-19 case rates before and after the initial vaccine rollout among health care personnel in Orange County, California.

## Introduction

By mid-April 2021, there were 133 million COVID-19 infections and 2.9 million associated deaths worldwide.^1^ Several vaccines offer hope to end the pandemic. COVID-19 mRNA vaccines provide 95% protection from symptomatic disease to date, but concern exists over asymptomatic infection and transmission risks in vaccinated individuals. Health care personnel (HCP) may be routinely tested, enabling assessment of vaccine impact on asymptomatic COVID-19 infection. We evaluated COVID-19 rates before and after HCP vaccination in Orange County, California, the sixth largest US county and one of the hardest hit by COVID-19 during the winter surge from 2020 to 2021.^1,2^

## Methods

This cohort study of HCP was conducted at University of California Irvine (UCI) Health, the sole academic medical center in Orange County, California, between November 1, 2020, and March 31, 2021 (21 weeks). We evaluated HCP COVID-19 cases, both symptomatic and asymptomatic, before and after initiating COVID-19 mRNA vaccination (Pfizer, Moderna) on December 16, 2020. Daily screening for CDC-defined COVID-19 symptoms (fever, fatigue, chills, myalgia, congestion, cough, loss of smell, shortness of breath, sore throat, nausea, diarrhea) and temperature were required for all HCP, with rapid nasopharyngeal testing if positive. Additionally, invitations for asymptomatic bilateral nares testing were provided weekly to randomly selected HCP (approximately 1000 [10%] per week until the week of December 13, 2020, and approximately 2500 [25%] weekly thereafter). All samples were processed using polymerase chain reaction (PCR) testing for SARS-CoV-2 at UCI Health’s Clinical Laboratory Improvement Amendments–certified laboratory. Depending on availability, COVID-19 reverse transcription–PCR testing was performed using Molecular Simplexa (DiaSorin), m2000 RealTime (Abbott), or Xpert Xpress SARS-CoV-2 (Cepheid). Demographic data (age, race, ethnicity) were obtained from occupational health and human resources records to assess for any differences in vaccine distribution.

Every HCP with COVID-19 was interviewed for symptoms assessment regardless of testing pathway (eg, symptomatic or asymptomatic). Rolling 7-day averages (means) of daily COVID-19 cases were calculated for UCI Health and countywide cases reported by public health.^2^ COVID-19 cases identified through asymptomatic testing were stratified by presence or absence of symptoms identified on interview.

This study followed the Strengthening the Reporting of Observational Studies in Epidemiology (STROBE) reporting guideline and was deemed exempt by the University of California institutional review board because it was non–human participants research. Data analysis was performed from November 2020 to March 2021 using Excel 2010 (Microsoft).

## Results

**Figure 1.**
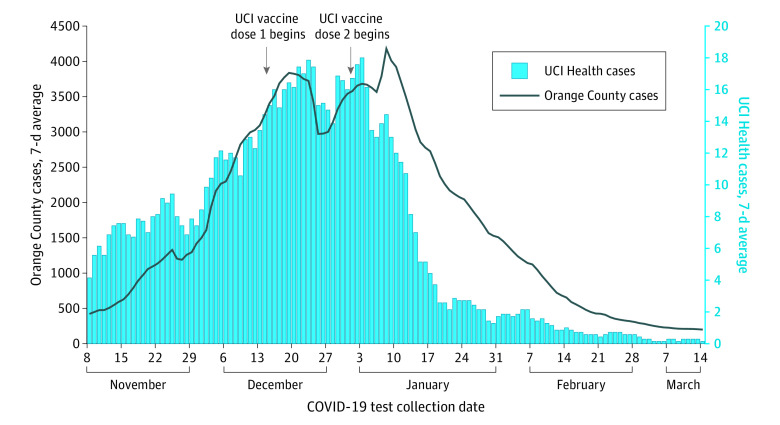
Daily Counts of COVID-19 Incident Cases Among Health Care Personnel in Orange County and University of California Irvine (UCI) Health COVID-19 cases among health care personnel declined after vaccination and in advance of countywide reductions. Data displayed as rolling 7-day averages (means) between November 8, 2020, to March 14, 2021.

Of 10 188 HCP included in the study, median (range) age was 38 (18-99) years; 6890 (61%) were female individuals, 2412 (19%) were Latino individuals, and 3339 (32%) were living in cities (top 5) with the highest COVID-19 burden in Orange County. During the winter surge, HCP COVID cases paralleled countywide prevalence, but diverged a week after second vaccine doses were initiated (Figure 1). Upon the start of vaccination, 58% of HCP (5683 of 9782 invited) received a first dose within 2 weeks, and 70% (7558 of 10748) within 4 weeks. After second doses began, daily HCP cases fell from 18 to 8 (55% reduction) after 1 week; to 3 (84% reduction) after 2 weeks, and to 1 (94% reduction) after 3 weeks.

**Figure 2.**
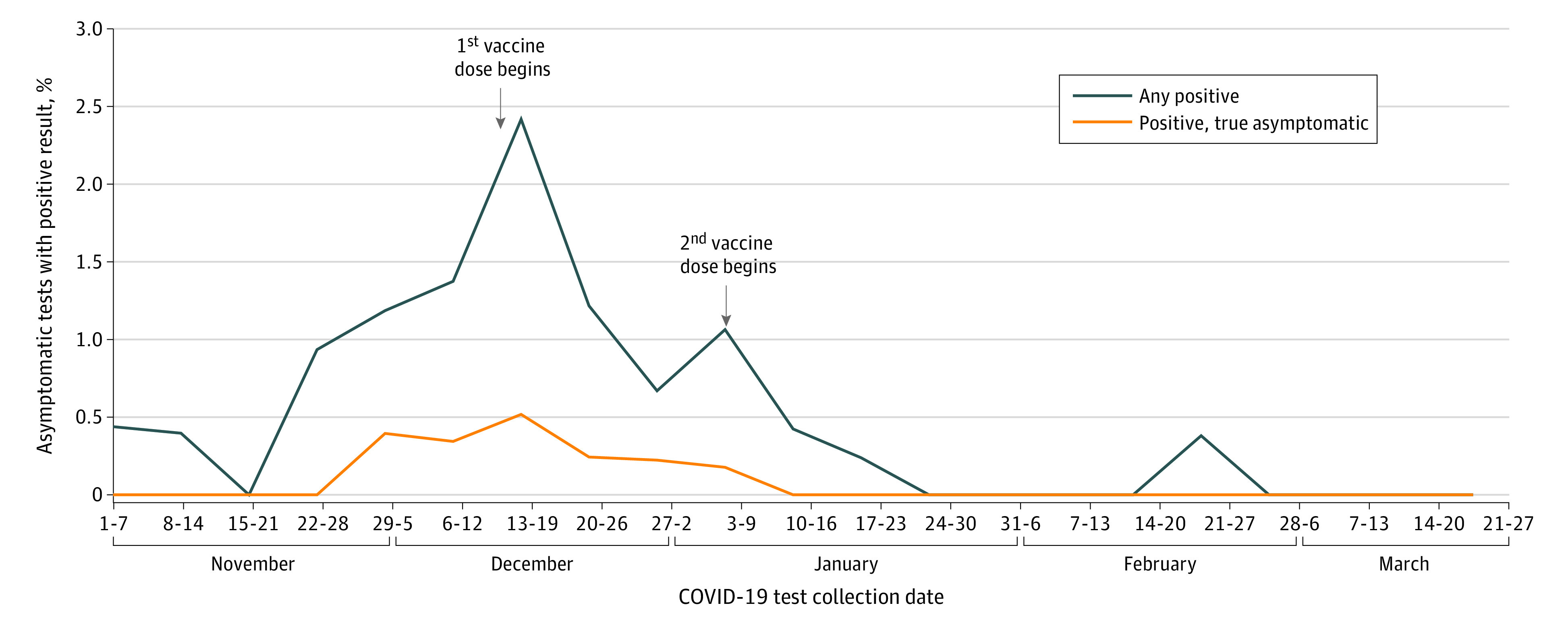
Percentage of Health Care Personnel Who Tested Positive for COVID-19 Using the Asymptomatic Testing Pathway Most health care personnel who tested positive using the asymptomatic testing pathway reported symptoms consistent with COVID-19 (81% [34 of 42]). Asymptomatic cases declined to 0 after vaccination.

Approximately 20% of invited HCP participated in weekly asymptomatic testing (approximately 250 HCP/week) when a random 10% were invited, and approximately 15% (approximately 500 HCP/week) participated when 25% were invited. Although symptomatic HCP were not to be tested using the asymptomatic pathway, most HCP with COVID-19 who were identified by asymptomatic testing (81% [34 of 42] within the 21-week study period) reported having symptoms at the posttest interview. Many dismissed symptoms in real-time as common ailments, but recognized them as COVID-19 symptoms in hindsight. Both symptomatic and asymptomatic COVID-19 disease identified through the asymptomatic testing pathway were reduced after vaccination (Figure 2).

## Discussion

This study found a rapid and sustained decline in both COVID-19 symptomatic and asymptomatic infections following HCP vaccination in a region experiencing high rates of COVID-19 disease nationally in the 2020 to 2021 winter season. HCP cases declined when our hospital was still in full surge with an active mobile field hospital and several outpatient areas commandeered for inpatient intensive care unit and non–intensive care unit beds. In addition, the decline in HCP cases in advance of a countywide decline is likely underestimated because 30% of essential workers are HCP who would have influenced countywide rates. Generalizability is limited by our single-center, regional experience. These findings are consistent with past experience with other viral respiratory infections, such as influenza and measles, in which vaccination reduces overall infection rates, viral shedding, and both symptomatic and asymptomatic transmission.^3,4,5,6^
